# Slums’ Access to and Coverage of Primary Health Care Services: A Cross-Sectional Study in Shiraz, a Metropolis in Southern Iran

**Published:** 2014-03

**Authors:** Hassan Joulaei, Azad R Bhuiyan, Mehrab Sayadi, Fariba Morady, Parvin Afsar Kazerooni

**Affiliations:** 1 HIV/AIDS Research Centre, Shiraz University of Medical Sciences, Shiraz, Iran;; 2 Department of Epidemiology and Biostatistics, Jackson State University, Mississippi, USA;; 3 Statistics and Information Technology Unit, Shiraz University of Medical Sciences, Shiraz, Iran;; 4 Shiraz Geriatric Research Center, Shiraz University of Medical Sciences, Shiraz, Iran;; 5 Control and Surveillance of Diseases Unit, Shiraz University of Medical Sciences, Shiraz, Iran

**Keywords:** Primary health care, Health services accessibility, Slums

## Abstract

**Background:** The United Nations has predicted that the population of slum dwellers will have grown from one billion people worldwide to 2 billion by 2030. This trend is also predictable in Iran. In the Iranian metropolis of Shiraz, more than 10% of the residents live in slum areas. There are several problems regarding the delivery of social services in these areas. The aim of this study was to evaluate slums dwellers’ access to and coverage of health care.

**Methods:** This cross-sectional face-to-face study included 380 household of slum dwellers via stratified random sampling. Demographics, accessibility of health services, coverage of health care, and route of receiving health services were recorded through interviews.

**Results:** Approximately, 21.6% of the households had no physical access to health centers. The coverage rate of family planning programs for safe methods was 51.4% (95% CI: 48.86-53.9%). Vaccination coverage among children under 5 years old was 98% (95% CI: 97-99%). Furthermore, 34% of pregnant women had not received standard health care due to a lack of access to health centers.

**Conclusion:** Limited access to health services along with inadequate knowledge of slum residents about health care facilities was the main barrier to the utilization of the health care in the slums.

## Introduction

Slum residency is an informal residency on the outskirts or even in the inner parts of a town. Slums have the least welfare and public services, including health services.^1^ The phrase “slum” is sometimes used in developing countries in order to elucidate the miserable living conditions of the residents of such areas. The percentage of urban residents living in slums decreased from 47% to 37% in developing countries between 1990 and 2005.^2^ Currently, one billion people live in slums^2^ worldwide and the United Nations (UN) has predicted that this figure will have risen to 2 billion by 2030.^3^ This will aggravate the current situation because only a few of these governments already have the financial resources to cope.^4^ For instance, Cairo receives 1000 new residents every week, which would definitely exacerbate job deficiency and hosing supplies in the capital of Egypt.^5^

Urban decay and high rates of poverty, illiteracy, and unemployment along with inadequate hygienic water, insufficient access to sanitation and other infrastructure, and poor structural quality of housing are very important indicators of slums.^6^ Slums are usually deemed "breeding grounds" for such social problems as crime, drug addiction, alcoholism, high rates of mental diseases, and suicide.^7^^,^^8^ It is predictable that by 2030, approximately 1.7 billion of the expected 3.93 billion urban dwellers in low-income and middle-income countries will be doubled.^9^ In this regard, Jolene Skordis-Worral and co-workers^10^ showed that the prevalence of self-reported morbidity in urban slum settings of India was 37.5%, which is higher than that of the regions with a well socioeconomic status.^10^ Also, in a panel study, researchers illustrated that urban slum prevalence exhibits a substantial impact on infant and child mortality across a large number of less-developed countries.^11^ In addition, in the slums of Nairobi and Kenya about 18% of the respondents reported being affected by HIV/AIDS.^12^ India has one of the largest urban populations in the world (28% of the total national population) and ranks among the top ten slum areas in the globe.^13^

The development of slums in Iran started in 1961 following a new rule of interaction between farmers and feudal employers. At the time, big cities were obliged to host hundreds of thousands of migrants from rural areas.^14^^,^^15^ In spite of remarkable achievements in the development of deprived areas, including hygienic drinking water distribution system, primary health care,^16^ and social services in Iran, the country is still far off the target of narrowing the gap between these areas. For instance, the poverty levels, unemployment rates, and maternal and neonatal mortality rates in slums are higher than those in urban areas.^17^ High-risk behavior, such as intravenous drug use (IDU), in these areas compared to other areas renders their residents more susceptible to communicable diseases such as HIV, hepatitis B or C, and other sexually transmitted diseases (STIs).^18^ Moreover, the distribution of health resources is not equitable and the present arrangements are unable to ensure the provision of basic health care services to all citizens.^19^ In Shiraz, a metropolis in the south of Iran, more than 10% of the total population (about 1.7 million) lives in slums.^20^

To the best of our knowledge, the present study is the first documented survey about the accessibility and coverage of primary health care services in Iranian slums. We conducted this study in order to evaluate the level of access to and coverage of primary health care services in the slum areas of Shiraz.

## Materials and Methods

The present study was a cross-sectional study, conducted in Shiraz slums, in order to assess the access to and coverage of its residents to primary health care services and the status of common diseases among them. The study was carried out from October 2009 to July 2010 and included a sample population of the households residing in the slums. Considering that 50% of the population of these areas has access to primary health care (experts’ opinion), confidence level at 95%, and margin of error at 5%, the sample size was calculated to be 380 according to the following formula:

$$\text{n}=\frac{{\text{z}}_{\text{1}-\frac{á}{\text{2}}}^{\text{2}}\text{p}\left(\text{1}-\text{p}\right)}{{\text{d}}^{\text{2}}}$$

The stratified cluster random sampling method was used in order to obtain a sample of 380 households. First, based on the municipality’s map, the marginal zones of Shiraz were specified and then, the sample number was determined based on the size of the population in each zone. According to their Zip Codes, the clusters were selected randomly and finally the participants were randomly drawn from all the households. 

The sampling unit was the household. Each participant was visited by a group of professionals at his/her home separately, and data gathering forms were filled out under the supervision of the group members. Household women were selected as respondents in the study because they are properly informed of the health situation of the family and also are readily available. Any reported diseases by the respondents had to be confirmed by medical documents.

Non-Iranians were also excluded. The study protocol was approved by the Institutional Review Board (IRB) and the Research Ethics Committee of Shiraz University of Medical Sciences. 

A structured data gathering form was used in order to obtain data from all the randomly selected participants by means of a face-to-face interview. The data gathering form was comprised of three parts: demographic data, including sex, age, and number of members in each household, accessibility of health services, and coverage of primary health care (such as children health care, family planning, maternal health care, common communicable and non-communicable diseases, and Pap smear for detecting cervical cancer); route of receiving health services whether public or private; and sources of health information.

As was mentioned in the Introduction, high-risk behavior is high among slums’ residents. Accordingly, the respondents’ knowledge of HIV/AIDS was assessed with a questionnaire containing nine close-ended questions (similar to those reflected in national or local surveys in Iran). These questions covered the categories of the definition of HIV/AIDS, mode of transmission, and routes of prevention. The questionnaire was validated by expert opinion and was pre-tested among 35 respondents. After data analysis, Cronbach's α was calculated to assess the internal consistency of the knowledge questions (α=0.63). The questions were answered using the options "Agree", "Disagree", and "I don't know". A total score for knowledge was obtained by adding the points given for each answer. For each correct answer one point, and for "I don't know" or any incorrect answer zero points were assigned. The sum makes up the total score, which ranged between 0 and 9. Scores >4.5 indicated acceptable knowledge and those <4.5 denoted poor knowledge.

Based on the definitions of the Iran's Ministry of Health and Higher Education, "excellent access" is less than a 10-minute walk to a health center; "acceptable access" is a 10 to 30-minute walk; and "inaccessibility" is more than a 30-minute walk.  

Maternal care is defined as at least six visits during pregnancy and two visits after delivery by health centers.^21^

Child health care refers to regular well-child check-up according to the program of the Iran's Ministry of Health and Higher Education.^21^

Contraception coverage is defined as the number of women in reproductive age who use safe methods of contraception divided by the total number of women between 15-59 years old.^21^

Vaccination coverage for children under 5 years is defined as an immunization schedule by which children under 5 years of age are protected against diphtheria, tetanus, pertussis, polio, measles, mumps, rubella, hepatitis B, and tuberculosis.^21^

Vaccination coverage in adults is defined as the coverage for diphtheria and tetanus vaccine among adults.^20^

Two major communicable diseases rife among slum dwellers are leishmaniasis and HIV/AIDS. As a result, researchers have selected both of them to assess the accessibility of the population to their health care.^22^ In this study, non-communicable diseases (according to the National Surveillance Program for Diabetes and Hypertension) and accessibility to health care for these diseases were assessed.^23^

SPSS software, version 17.0 (SPSS Inc., Chicago, Ill., USA) was used in order to analyze the data statistically. The results are expressed as mean±SD and proportions as appropriate. The Chi-squared test was used to compare the proportions between different groups. A two-sided P<0.05 was considered statistically significant. 

## Results

Overall, 372 inhabitants responded to our survey (response rate=98%). The mean age of the participants was 29.6±7.4 (ranging from 19 to 54) years. The family size of the target population was 4.5±1.7. The average accessibility time was 17.6±6.2 minutes, and about 21.6% (80 families) of the households had no physical access to health centers. The coverage rate of family planning programs was 66.4% (95% CI: 61.6-71.19%), whereas about 15% of the respondents used natural methods such as withdrawal. Table 1 demonstrates the contraceptive prevalence rate of each method among the slums' dwellers compared with the rural and urban populations of the Fars Province.

**Table 1:** Frequency of contraceptive methods among the slums' residents in comparison with the urban and rural areas of the Fars Province (2009-2010)

** Location** **Contraceptive Method**	**Suburban residents**			**Rural areas (20)**		**Urban areas (20)**	
	**N**		**%**	**N**	**%**	**N**	**%**
Pill	71	19.15		55948	24.40	243	18.35
Condom	39	10.42		25543	11.15	208	15.72
Tuba ligation	43	11.57		43773	19.10	191	14.44
Vasectomy	6	1.68		5623	2.50	57	4.27
Injection	15	3.94		10755	4.70	47	3.52
IUD	17	4.49		9388	4.1	81	6.15
Total of safe methods	191	51.25		151030	65.95	827	62.50
Natural methods	56	15.15		17890	7.80	1084	19.4
Total of methods	247	66.4		168920	73.75	1078	81.9
Total of samples	372	100		228982	100	1323	100

Only 46% of the eligible women were screened by the Pap smear test; in addition, 62% of the uncovered women reported inaccessibility to such screening tests. In 34% of the cases who received no standard health care services during pregnancy, limited accessibility to the health centers was the main cause. Thirty-six percent of the women had not received postpartum care because of limited accessibility and unawareness. 

Eighty-eight percent of children under 8 years of age were covered by public health services, and vaccination coverage for this age group was estimated to be 98% (95% CI: 97-99%). The vaccination coverage of men above 16 years old was 49%, while it was 78% for women of  the same age. According to the participants’ response, 7.2% (95% CI: 6.4-8%) and 10% (95% CI: 9.08-10.91%) of the slums' residents over 15 years were diabetic and hypertensive, respectively. Also, 18% of hypertensive individuals were not under health care coverage because of inaccessibility and 55% also referred to private health centers, irregularly. About 16.3% of the participants (95% CI: 14.91-17.68%) over 15 years old were smokers. Approximately, 51% of the respondents mentioned that they had at least one addicted family member, 10% of them being intravenous drug abusers. 

About 8.5% of the households' members had been exposed to leishmaniasis; of which 35.7% and 28.6% had referred to public and private health centers, respectively. Four (1.1%) respondents reported that they had one member infected by HIV. Approximately, 18.6% of the respondents had poor knowledge about the definition of HIV/AIDS and its routes of transmission and prevention. Our findings revealed that the most prevalent health information source in the slums was the radio and the least prevalent one was health care centers' staff. 

## Discussion

Health care services in slums and the health status of their residents have become a public health challenge in the current century. The Iranian metropolis of Shiraz has a good health care network in that it provides health care access to more than 85% of its whole population.^22^ Nevertheless, many dwellers of the slums of this city are deprived from basic health care services.^24^ It is clear that the current locations of the health care centers in the slums are not compatible with standard protocols. The problem is compounded by the fact that there is no reliable information and evidence on the exact health status of the residents of these regions. In this study, we found out that 21.6% of the slums were not covered by health care services; this rate is much higher than that in rural areas (less than 5%).^22^ Studies among slums in India^25^ and Africa^26^ have also pointed out that accessibility is a very important contributing factor in the utilization of health care services among slum dwellers.^25^^,^^26^ According to our study, the coverage of contraception in the rural and urban areas of Fars Province was roughly 66% and 63%, respectively, while this figure in the slums stood at 51%, which is significantly lower than the rate in the rural areas (P<0.001).^21^ Moreover, the contraceptive prevalence rates of all methods among married women of reproductive age (15-49) in Iran is 73%,^27^ as opposed to 66.4% in our study. Also in our study, 19.15% of the participants were on oral contraceptives, showing that this method was the most commonly used, while the least common one was intramuscular injection of Medroxyprogesterone Acetate (3.94%). Approximately, 28% of the women of reproductive age in our study were not using any method of contraception due to a lack of accessibility to public health centers.

Our findings demonstrated that 28% of the women had not registered and followed family planning programs. This could be due to difficult access to health care centers, which is compatible to other studies.^25^^,^^26^ According to evidence, female dwellers of slums have much higher fertility rates than their urban peers; moreover, in many regions of Asia and Africa, in the slums, fertility rates are similar to those of rural areas.^9^ The low coverage of family planning programs in the slums of Shiraz along with immigration from rural to urban areas has led to high rates of population growth and consequently increased incidence rates of antisocial and risky behaviors, addiction, STDs, and other psycho-socio-medical hazards.^28^ In our study, over 34% of the pregnant women were deprived from maternal and postpartum health care. These statistics, when juxtaposed against the total country rates (27.5% and 15.6% for maternal and postpartum care, respectively)^29^are indeed disconcerting. There could be two explanations: lack of general health knowledge among the slums’ women^30^ and poor accessibility to health care centers.^31^

Based on the Iranian National Vaccination Program, all children below the age of 6 must be vaccinated against tuberculosis, diphtheria, tetanus, pertussis, hepatitis B, poliomyelitis, and measles. Be that as it may, the results of our study indicated that the rate of the complete coverage of the Iranian National Vaccination Program was 98% in the slums. Regarding the Urban Heart Survey in Tehran, vaccination coverage among children over the age of 13 years in six districts out of twenty-three was below 80%. The socioeconomic indicators of the survey showed that most of those mentioned districts had a low socioeconomic status.^32^ A comparison between the results of these two studies suggest that vaccination coverage in Shiraz’s slums is higher than Tehran’s low socioeconomic areas. There is also a low frequency of hepatitis B vaccination among the youth (52%) in comparison with the coverage of rural areas (76.8%)^20^ (P<0.001). In addition, the overall health care coverage of the children of the slums was 83%, which is lower than that in the rural areas (98%).^22^

Our findings showed a higher prevalence rate of adult smokers in these areas in comparison with the rate of the whole country (16.3% and 12.3%, respectively).^23^ Nonetheless, according to the World Health Organization (WHO), the average proportion of adult smokers in the Eastern Mediterranean region is 19%.^33^

Although over 90% of patients with diabetes mellitus and/or hypertension have access to health care and utilize routine and programmed visits by family physicians in rural areas, this rate is less than 83% in the slums.^22^

One of the most important health issues in slums is the prevalence of communicable diseases.^9^ Higher incidence and prevalence rates of HIV/AIDS in these areas endorse this fact. A comparison of our respondents' knowledge about the transmission and prevention routes of HIV/AIDS with other studies indicated higher poor knowledge rates in our study population.^34^

In two Nairobi slums, about 1% of the respondents reported being infected with HIV,^12^ whereas this figure in our study was less than 0.24%. However, this rate is also significantly higher than the rate of the total country (0.032%).^35^

In a study among the slum population in Nairobi, the investigators concluded that the Integrated Management of Child Illnesses (IMCI) program must be free of charge to the urban residents in order to increase health care seeking and improve survival of children.^36^ In the urban and rural areas of Iran, health care services are free of charge for all people. It seems that the main problem in Iran is that these centers are not readily accessible to the residents.

Our findings showed that the radio (52%), followed by television (32%), was the main source of health information provision for the respondents. It was remarkable that health care centers' staff were responsible for delivering health information of only 3% of the respondents.        Establishing new health care centers in slum areas, augmenting the quality of medical services in health care centers, and elevating health knowledge among slum dwellers constitute three major strategies that should be adopted in order to combat this challenge.  

## Conclusion

Primary health care service is essential for all different social strata. However, access to and coverage of health care is dissimilar in the different areas of the Iranian province of Fars. Several factors are involved in the genesis of this problem. Low accessibility to and shortage of perfect coverage of primary health care in slums areas along with inadequate health knowledge of their residents deprive the majority of these slums’ residents of good health. 
